# Mitochondrial regulation of cellular senescence heterogeneity

**DOI:** 10.3389/fcell.2026.1866640

**Published:** 2026-08-12

**Authors:** Minseo Ahn, Sung-Jin Yoon, Jae Ho Seo

**Affiliations:** 1 Department of Biochemistry, Wonkwang University School of Medicine, Iksan, Republic of Korea; 2 Sarcopenia Total Solution Center, Wonkwang University School of Medicine, Iksan, Republic of Korea; 3 Institute of Wonkwang Medical Science, Wonkwang University School of Medicine, Iksan, Republic of Korea; 4 Environmental Disease Research Center, Korea Research Institute of Bioscience and Biotechnology (KRIBB), Daejeon, Republic of Korea; 5 Department of Functional Genomics, KRIBB School of Bioscience, University of Science and Technology (UST), Daejeon, Republic of Korea

**Keywords:** cellular senescence, metabolic reprogramming, mitochondrial dysfunction, mitophagy, reactive oxygen species (ROS), senescence heterogeneity, senescence-associated secretory phenotype (SASP), senolytics

## Abstract

Cellular senescence is a stable cell-cycle arrest program accompanied by extensive metabolic remodeling and acquisition of a senescence-associated secretory phenotype (SASP). Emerging evidence indicates that senescence is not a uniform endpoint but a heterogeneous spectrum of cell states shaped by the nature of the initiating stimulus. Mitochondria have recently emerged as central regulators of this heterogeneity by integrating metabolic, redox, and inflammatory signaling. Senescent cells share common mitochondrial features-including increased mitochondrial mass, elevated reactive oxygen species (ROS), impaired mitophagy, and altered metabolic programs-yet distinct senescence subtypes exhibit unique mitochondrial adaptations. Replicative senescence is governed by a telomere–mitochondria feedback loop, whereas stress- and oncogene-induced senescence involve rapid mitochondrial stress responses and stimulus-specific metabolic rewiring. Therapy-induced senescence further introduces context-dependent mitochondrial dependencies that influence therapeutic resistance and senolytic vulnerability. In this review, we synthesize current understanding of mitochondrial regulation across senescence subtypes and highlight how mitochondrial dysfunction actively drives senescence heterogeneity. We further discuss emerging therapeutic strategies that exploit mitochondrial vulnerabilities to selectively modulate or eliminate senescent cells. Understanding mitochondrial control of senescence heterogeneity provides a conceptual framework for developing precision interventions in aging and cancer.

## 1 Introduction

Cellular senescence is a stable state of cell-cycle arrest induced by diverse intrinsic and extrinsic stressors, including telomere attrition, oncogene activation, oxidative stress, mitochondrial dysfunction, and DNA damage (Campisi, 1997; Cristofalo et al., 2004; Hernandez-Segura et al., 2018; Gorgoulis et al., 2019; Campisi, 2013). Senescent cells exhibit characteristic phenotypes including permanent growth arrest and the senescence-associated secretory phenotypes (SASPs), consisting of chemokines, growth factors, and matrix-remodeling factors (Basisty et al., 2020; Coppé et al., 2010). Depending on the nature, intensity, and duration of the inducing stimulus, senescent cells may display distinct transcriptional, metabolic, and secretory profiles, highlighting the functional heterogeneity of senescence programs (Hernandez-Segura et al., 2018; Gorgoulis et al., 2019). This diversity is reflected in differences in metabolic rewiring, chromatin organization, secretory profiles, and interactions with the immune microenvironment. Replicative senescence (RS) arises from progressive telomere shortening and activation of persistent DNA damage responses that enforce permanent cell-cycle arrest (Campisi, 1997; Cristofalo et al., 2004). Stress-induced premature senescence (SIPS) is driven by acute stressors such as oxidative damage, ultraviolet radiation, or chemical insults. Oncogene-induced senescence (OIS) results from aberrant mitogenic signaling triggered by oncogenes such as RAS or BRAF, functioning as an intrinsic barrier to malignant transformation. Therapy-induced senescence (TIS) occurs when anticancer treatments―including chemotherapy and radiation―induce senescence-like growth arrest in tumor and surrounding cells, producing both beneficial anti-proliferative effects and detrimental consequences through sustained SASP activity. Each subtype exhibits distinct molecular and phenotypic characteristics, including differences in metabolic reprogramming, chromatin organization, SASP composition, and immune surveillance profiles.

Mitochondria have emerged as critical regulators of cellular senescence. Beyond their classical role in ATP production, mitochondria integrate metabolic signaling, reactive oxygen species (ROS) generation, calcium homeostasis, and apoptotic control. Mitochondrial dysfunction is widely recognized as a hallmark of aging and is consistently observed in senescent cells (Miwa et al., 2022; Wei et al., 2025; Correia‐Melo et al., 2016). However, emerging evidence suggests that not all mitochondrial alterations observed during senescence necessarily represent irreversible dysfunction. Depending on cellular context and senescence-inducing stimuli, certain mitochondrial changes may reflect adaptive metabolic remodeling that supports establishment and maintenance of the senescent phenotype (Marmisolle et al., 2025).

Recent studies further suggest that mitochondria play a pivotal role in shaping senescence heterogeneity. Distinct senescence-inducing stimuli generate unique mitochondrial states, characterized by differences in mitochondrial dynamics, metabolic programs, redox balance, and inflammatory signaling outputs. These mitochondria-dependent variations ultimately influence key functional properties of senescent cells, including SASP composition, immune surveillance, and susceptibility to therapeutic interventions. Despite extensive studies on mitochondrial dysfunction in aging, a unifying framework that explains how mitochondria actively generate senescence heterogeneity across distinct contexts remains lacking.

In this review, we summarize the common mitochondrial features shared across senescent cells and discuss the subtype-specific mitochondrial adaptations associated with replicative, stress-induced, oncogene-induced, and therapy-induced senescence. We further highlight how mitochondrial regulation contributes to the functional heterogeneity of senescent cells and discuss emerging therapeutic strategies that exploit mitochondrial vulnerabilities to modulate or eliminate senescent cells. By integrating mitochondrial biology with senescence heterogeneity, this review aims to provide a conceptual framework for developing mitochondria-targeted interventions in aging and cancer.

## 2 Mitochondrial features of senescent cells

Senescent cells frequently exhibit mitochondrial alterations characterized by reduced oxidative phosphorylation (OXPHOS) efficiency, changes in mitochondrial membrane potential, and remodeling of mitochondrial ultrastructure (Gorgoulis et al., 2019; Basisty et al., 2020; Coppé et al., 2010; Hernandez-Segura et al., 2018). Alterations in mitochondrial cristae architecture have been reported in several senescence models, although the extent and characteristics of these changes appear to vary depending on cell type and senescence-inducing stimuli (Campisi, 2013; Varesi et al., 2022; Tak et al., 2024; Xiong et al., 2025). A central driver of this dysfunction is impaired mitophagy, which prevents the clearance of damaged mitochondria and leads to their progressive accumulation (Kataura et al., 2025; Diniz et al., 2024). Under physiological conditions, the PINK1-Parkin pathway serves as a central mechanism of selective mitophagy, whereby damaged mitochondria are recognized and targeted for lysosomal degradation to maintain mitochondrial quality control. Suppression of the PINK1–Parkin pathway, frequently observed in senescence models, result in sustained reactive oxygen species ROS production, mitochondrial DNA (mtDNA) instability, and metabolic disruption. Notably, the extent of mitophagy impairment varies across senescence subtypes―gradual decline in replicative senescence versus acute disruption in stress-induced senescence―contributing to heterogeneity in mitochondrial integrity and SASP intensity. Restoration or enhancement of mitophagy alleviates senescence-associated phenotypes in multiple experimental systems, highlighting mitophagy as a key regulatory node linking mitochondrial quality control to senescence heterogeneity (Ma et al., 2021; Kelly et al., 2024). Paradoxically, despite functional decline, senescent cells often exhibit increased mitochondrial mass, likely reflecting compensatory biogenesis driven by mTORC1-PGC-1 signaling (Miwa et al., 2022; Lee et al., 2002; Nacarelli et al., 2016).

Accumulation of dysfunctional mitochondria leads to persistent oxidative stress. Impaired electron transport chain (ETC) activity promotes electron leakage and superoxide production, which is further exacerbated by increased ER–mitochondria contact, mitochondrial Ca^2+^ overload, and iron accumulation (Miwa et al., 2022; Chapman et al., 2019; Ahumada-Castro et al., 2021; Seo et al., 2008). Concurrently, antioxidant system—including Nrf2-dependent pathways—is compromised, resulting in sustained ROS elevation (Varesi et al., 2022; Ebrahimirad et al., 2025). Chronic ROS not only induce the DNA damage response (DDR) but also amplifies the senescence-associated secretory phenotype (SASP) through activation of NF-κB and C/EBPβ signaling (Korolchuk et al., 2017; Martini and Passos, 2023; Zia et al., 2022; Frey et al., 2025).

Mitochondrial dysfunction in senescence is further accompanied by profound alterations in mitochondrial dynamics (Zhan et al., 2013; Tilokani et al., 2018). Senescent cells frequently exhibit altered mitochondrial fusion and fission varies according to the senescence-inducing stimulus and cellular context. Hyperfused, elongated, and highly interconnected mitochondrial networks have been observed in replicative, oncogene-induced, and therapy-induced senescence models (Liu et al., 2020; Seo et al., 2010), whereas excessive mitochondrial fission has also been reported in specific stress-induced senescence settings (Michikawa et al., 1999; Pi et al., 2019). This fusion-dominant state exerts dual effects: it impairs mitophagy-mediated clearance of damaged organelles while simultaneously preserving mitochondrial membrane potential and limiting excessive fragmentation-induced apoptosis (Ziegler et al., 2015; Lee et al., 2007; Ikeda et al., 2014; Chen et al., 2023; Lacombe and Scorrano, 2024; Mai et al., 2010; Zorov et al., 2014; Wang et al., 2025). Thus, mitochondrial hyperfusion represents both a contributor to mitochondrial dysfunction and compensatory adaptation that supports cell survival. These structural and functional alterations are tightly coupled to metabolic rewiring. Reduced OXPHOS efficiency drives a compensatory shift toward glycolysis, while declining NAD^+^ levels impair sirtuin activity (SIRT1/3/6), further exacerbating mitochondrial dysfunction and genomic instability (Ziegler et al., 2015; Wiley and Campisi, 2021). In parallel, altered fatty acid oxidation (FAO) increases acetyl-CoA availability, linking mitochondrial metabolism to epigenetic regulation and SASP production. NAD^+^ depletion also sensitizes the mitochondrial permeability transition pore (mPTP), reinforcing a feedforward loop that promotes senescence progression (Chini et al., 2024; Xie et al., 2020; Rottenberg and Hoek, 2021; Rottenberg and Hoek, 2017; Yamauchi et al., 2024; Liu et al., 2023).

Mitochondrial dysfunction additionally reshapes the epigenetic landscape. Changes in mtDNA methylation and disruption of epigenetic enzyme activity by mitochondrial ROS contribute to transcriptional reprogramming that stabilizes the senescent state (Bianchessi et al., 2016; Wang et al., 2021).

A critical emerging feature is the release of mitochondria-derived signaling molecules. Mitochondrial DNA (mtDNA), released into the cytosol or extracellular space under stress conditions, acts as a damage-associated molecular pattern (DAMP) that activates the cGAS–STING pathway and drives pro-inflammatory SASP signaling (Iske et al., 2020). Variations in mtDNA release and stability across senescence subtypes contribute to differences in inflammatory outputs and immune interactions (Victorelli et al., 2023; Ramini et al., 2024). In addition, mitochondrial retrograde signaling—mediated by ROS and stress-activated pathways—promotes cytoplasmic chromatin fragment formation and further amplifies SASP responses (Miwa et al., 2022; Ramini et al., 2024; Victorelli et al., 2023; Vizioli et al., 2020; Bianchessi et al., 2015).

Collectively, these findings indicate that mitochondrial alterations are not merely passive consequences of senescence but represent a dynamic regulatory platform that integrates metabolic, redox, and inflammatory signaling to shape the phenotype and functional heterogeneity of senescent cells. Importantly, some mitochondrial changes may reflect adaptive metabolic remodeling that supports establishment and maintenance of the senescent state, whereas others contribute directly to mitochondrial dysfunction and cellular decline (Hernandez-Segura et al., 2018; Wiley and Campisi, 2021; Valerio et al., 2021; Hara et al., 2013). The major mitochondrial features, metabolic adaptations, regulatory pathways, and therapeutic vulnerabilities associated with distinct senescence subtypes are summarized in Table 1.

**TABLE 1:** Mitochondrial features across senescence subtypes.

Senescence type	Primary trigger	Mitochondrial mass and structure	ROS	Metabolic reprogramming	Key regulatory signaling	Therapeutic vulnerabilities	Source
Replicative senescence (RS)	Telomere shortening during repeated cell division	Variable mitochondrial content and mass depending on cell type. Progresses to hyperfused networks	Gradual increase with stochastic mitochondrial ROS variability. ROS accelerates telomere shortening	Shift toward lipid metabolism, including enhanced fatty acid uptake and oxidation. Declining NAD^+^ levels	Bidirectional telomere-mitochondria feedback loop. p53-mediated PGC-1 suppression. NAMPT-NAD^+^-SIRT1 axis downregulation	NAD^+^ restoration (e.g., NMN, NAM). Interventions reducing oxidative stress or mild mitochondrial uncoupling	55,57,61
Stress-induced premature senescence (SIPS)	Acute stressors such as oxidative damage, UV radiation, hypoxia, and chemical agent	Rapid disruption of membrane potential. Characterized by excessive mitochondrial fission and suppressed mitophagy	Acute ROS burst followed by sustained oxidative stress. Self-amplifying cycle of mtDNA damage and ROS.	Impaired bioenergetics. Altered NAD^+^ metabolism in MiDAS.	p53-p21 and p16-pRb pathways activation. cGAS-STING activation via cytosolic DNA.	Low-dose resveratrol for adaptive ROS signaling. Mitochondrial transplantation	82,84,95
Oncogene-induced senescence (OIS)	Oncogene signaling (e.g., RAS, BRAF)	Early mitochondrial activation with increased mass and mtDNA. Progresses to a hyperfused and elongated network	Early NOX4-dependent ROS surge. Sustained mitochondrial ROS production	Highly context-dependent. Shows enhanced FAO/OXPHOS in some models or glycolytic shifts in others	NOX4-p22 complex drives initial DDR. Calcium signaling via MCU and SCN9A stabilizes mitotic arrest	Targeting specific metabolic dependencies based on the context (e.g., OXPHOS vs. glycolysis)	59,97,104
Therapy-induced senescence (TIS)	Anticancer treatments include chemotherapy, radiotherapy, and targeted therapies	Early mitochondrial volume increase followed by lipid vesicle accumulation. Shifts toward hyperfused networks	Elevated ROS levels depending on the specific therapy used (e.g., doxorubicin, cisplatin)	Highly heterogeneous bioenergetics. Frequent shift toward glycolysis with AMPK activation and lactate secretion	“Mitochondrial memory” alters apoptotic priming. Dysregulated fission-fusion balance	Highly vulnerable to senolytics due to anti-apoptotic dependencies. MitoTam targets low ANT2 levels	111,114

Overview of mitochondrial features across major senescence subtypes (RS, SIPS, OIS, and TIS), including triggers, mitochondrial alterations, ROS dynamics, metabolic reprogramming, regulatory pathways, and therapeutic vulnerabilities. While common mitochondrial dysfunction is observed, each subtype exhibits distinct adaptations contributing to senescence heterogeneity

Although aging and cellular senescence share multiple mitochondrial phenotypes, findings derived from aged tissues should not be considered equivalent to observations obtained directly from cellular senescence models. Aging reflects the cumulative influence of numerous systemic and tissue-level processes, whereas senescence represents a specific cellular stress-response program. Therefore, conclusions extrapolated from aging studies should be interpreted cautiously when discussing senescence-associated mitochondrial alterations.

**FIGURE 1:**
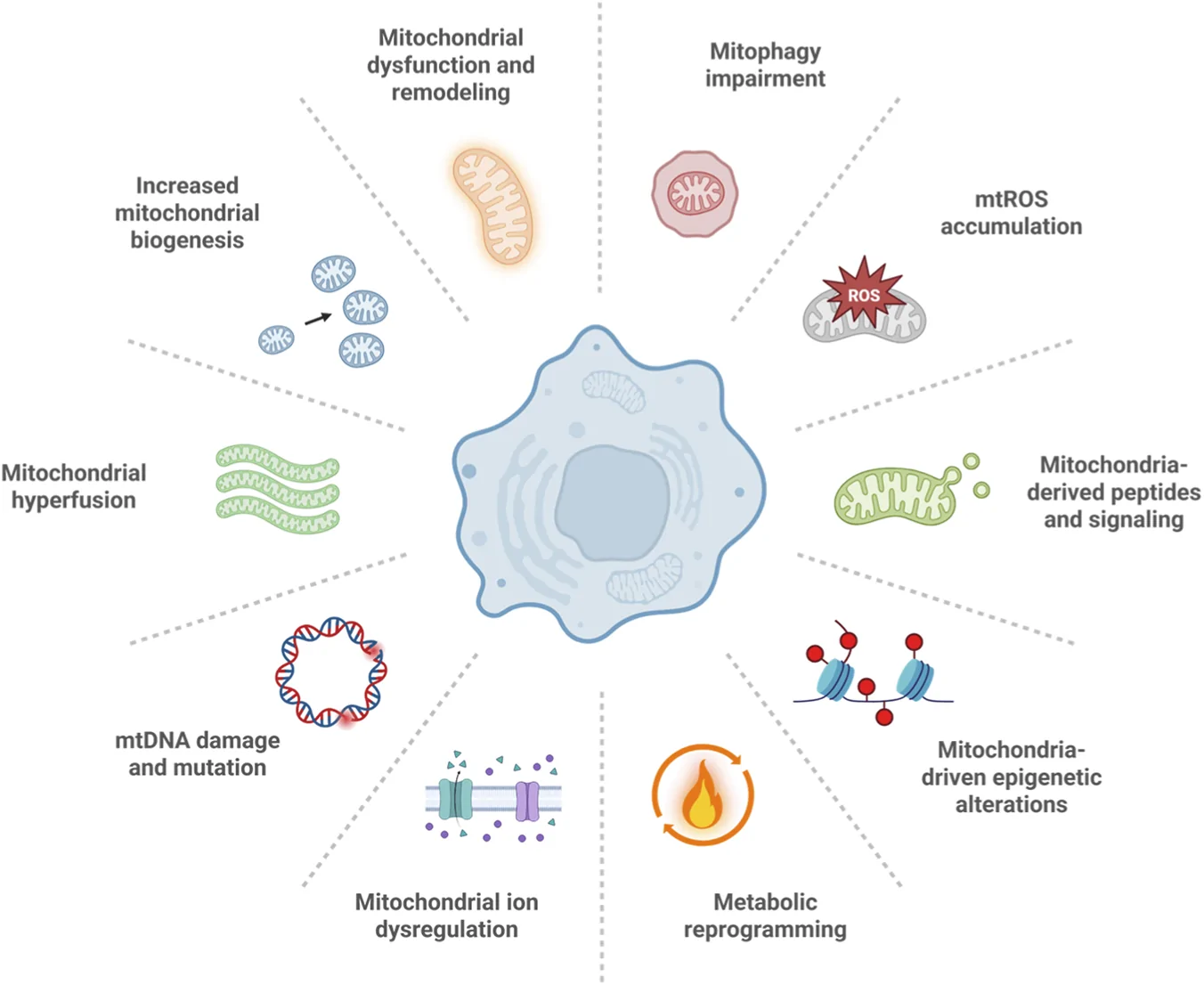
An overview of the general mitochondrial features of cellular senescence. Mitochondria integrate metabolic, redox, and inflammatory signaling to shape senescence heterogeneity. Distinct stimuli—including replicative stress, environmental stress, oncogenic signaling, and therapeutic interventions—induce subtype-specific mitochondrial alterations such as changes in dynamics, impaired mitophagy, increased mitochondrial mass, and elevated ROS. These mitochondrial changes regulate downstream processes including DNA damage response, metabolic rewiring, and SASP signaling, ultimately determining functional diversity and therapeutic vulnerability of senescent cells.

An overview of the general mitochondrial features of cellular senescence is summarized in Figure 1.

## 3 Mitochondria in replicative senescence

Replicative senescence (RS), also known as the Hayflick limit, is a permanent growth arrest triggered by progressive telomere shortening during repeated cell division. While RS shares common mitochondrial features with other senescence types—such as mitochondrial dysfunction and hyperfused networks—it is uniquely driven by a telomere-dependent mechanism that shapes its distinct mitochondrial phenotype (Passos et al., 2007a; Tak et al., 2024; Kipling, 2001; Shawi and Autexier, 2008; Muraki et al., 2012; Bernadotte et al., 2016).

Telomere attrition activates a persistent DNA damage response that stabilizes p53, which in turn represses PGC-1α/β, key regulators of mitochondrial biogenesis. This results in cell-type-dependent alterations in mitochondrial content, with some cell types exhibiting reduced mtDNA levels, while others―particularly fibroblasts―show increased mitochondrial mass and mtDNA copy number (Lee et al., 2002; Zheng et al., 2019; Sahin and DePinho, 2012; Schmid et al., 2019; Casorati et al., 2025; Lee et al., 2020; Kaplon et al., 2013; Finkel, 2011).

A defining feature of RS is its stochastic nature, largely driven by variability in mitochondrial dysfunction. Even within clonal populations, cells exhibiting higher mitochondrial ROS levels display accelerated telomere shortening, increased telomeric DNA damage, and earlier entry into senescence. Modulating mitochondrial ROS—for example, through mild uncoupling—can delay telomere dysfunction and extend replicative lifespan, highlighting mitochondrial ROS as a key determinant of RS heterogeneity at the single-cell level (Passos et al., 2007a; Passos et al., 2007b).

A critical bidirectional feedback loop links telomere dysfunction and mitochondrial dysfunction in RS. Mitochondrial ROS accelerates telomere shortening and genomic instability, while dysfunctional telomeres further enhance mitochondrial ROS production. Telomeres are particularly sensitive to oxidative damage due to inefficient repair capacity, making them effective sensors of mitochondrial stress. In addition, ROS-induced nuclear export of telomerase reverse transcriptase (TERT) reduces nuclear telomerase activity, further exacerbating telomere erosion. This self-reinforcing cycle drives progressive cellular aging, while interventions that reduce oxidative stress or restore mitochondrial function can mitigate telomere attrition (Passos et al., 2007b; Chung et al., 2008; Estrada et al., 2013; Haendeler et al., 2004; Liu et al., 2002; Michikawa et al., 1999).

Mitochondrial dysfunction in RS also triggers a retrograde signaling response that remodels nuclear gene expression. Loss of mitochondrial membrane potential reduces Ca^2+^ buffering capacity, leading to elevated cytosolic Ca^2+^ levels and activation of transcriptional programs such as CREB-dependent p21 induction, reinforcing cell cycle arrest. This retrograde response further coordinates metabolic adaptation, stress signaling, and survival pathways (Ziegler et al., 2015; Passos et al., 2007a).

NAMPT is the rate-limiting enzyme in the NAD^+^ salvage pathway, while SIRT1 is an NAD^+^-dependent deacetylase that regulates mitochondrial biogenesis, stress responses, and metabolic homeostasis. The NAMPT–NAD^+^–SIRT1 axis represents another central regulatory pathway in RS. Progressive downregulation of NAMPT reduces NAD^+^ levels and SIRT1 activity, promoting mitochondrial dysfunction and senescence progression. Conversely, restoration of NAD^+^ through metabolic intermediates such as NMN or NAM can delay replicative senescence and improve mitochondrial function, highlighting this axis as a key metabolic checkpoint (Miwa et al., 2022; Pi et al., 2019).

Consistent with broader senescence-associated metabolic reprogramming, RS cells shift toward lipid metabolism, including enhanced fatty acid uptake and increased fatty acid oxidation (FAO). This metabolic rewiring links mitochondrial dysfunction to epigenetic regulation, as altered metabolite availability influences chromatin structure and gene expression. For instance, reduced citrate export in mesenchymal stem cells decreases histone acetylation, promoting chromatin compaction and reinforcing the senescent state (Lee et al., 2020; Lizardo et al., 2017; Seok et al., 2020; Zhang et al., 2024).

Collectively, these findings define replicative senescence as a mitochondria–telomere coupled system, in which stochastic mitochondrial dysfunction, redox imbalance, and metabolic regulation converge to determine the onset and heterogeneity of the senescent state.

## 4 Mitochondria in stress-induced premature senescence

Stress-induced premature senescence (SIPS) arises rapidly in response to diverse environmental and intracellular stressors, in contrast to the gradual progression of replicative senescence. Across SIPS models, mitochondria function as both primary targets and active drivers of senescence, coordinating oxidative stress, DNA damage responses, and stable cell cycle arrest.

Oxidative stress is a central inducer of SIPS. Hydrogen peroxide (H_2_O_2_), one of the most widely used triggers, rapidly disrupts mitochondrial membrane potential, alters mitochondrial mass, and activates the p53–p21 and p16–pRb pathways (Abe et al., 2025). Mitochondria simultaneously serve as major sources of ROS, and sustained oxidative exposure leads to mtDNA damage, further amplifying mitochondrial dysfunction and ROS production in a feedforward manner (Pieńkowska et al., 2020; Duan et al., 2005). Other oxidants, including tert-butyl hydroperoxide (tBHP) and vitamin K_3_, similarly induce SIPS by impairing mitochondrial bioenergetics, increasing ROS, and collapsing membrane potential (Wedel et al., 2020; Loor et al., 2010). Metabolic stressors such as high glucose further exacerbate mitochondrial ROS production, promote excessive mitochondrial fission, and suppress mitophagy, collectively driving senescence (Tan et al., 2025).

Oxygen imbalance also critically influences mitochondrial dysfunction in SIPS. Hyperoxia elevates mitochondrial ROS, promotes mitochondrial fission, and induces ER–mitochondria stress, leading to activation of the p53/p21 and p16/Rb pathways and increased secretion of pro-inflammatory and pro-fibrotic SASP factors such as IL-6, IL-8, and PAI-1 (Koloko et al., 2025). In contrast, mild physiological hypoxia (1%–5% O_2_) can suppress senescence by limiting ROS production, preserving mitophagy, and inhibiting mTORC1-driven geroconversion through stabilization of HIF-1α (Leontieva et al., 2012; Welford et al., 2006). However, chronic or intermittent hypoxia may promote senescence via reoxygenation-induced ROS bursts, excessive mitochondrial fragmentation, and dysregulated HIF-1α signaling (Polonis et al., 2020; Wang et al., 2022).

DNA damage–inducing agents represent another major class of SIPS triggers, tightly linked to mitochondrial dysfunction. Doxorubicin induces senescence through mtROS accumulation, mitochondrial permeability transition pore (mPTP) opening, impaired mitophagy, and mtDNA release (Foglio et al., 2023; Xiong et al., 2025; Abdeahad et al., 2025). Bleomycin promotes cytoplasmic DNA accumulation and activates cGAS–STING signaling, reinforcing inflammatory senescence phenotypes (Bang et al., 2019). UV radiation induces a self-amplifying cycle of mtDNA damage and ROS production, resulting in mitochondrial dysfunction, increased mitochondrial mass, and dependence on PINK1–Parkin–mediated mitophagy (Yuan et al., 2025; Valerio et al., 2021; Cavinato et al., 2024). Ionizing radiation further contributes by impairing electron transport chain function, elevating mtROS, and altering nuclear epigenetic regulation (Lafargue et al., 2017; Szumiel, 2015).

Importantly, direct mitochondrial perturbation can itself initiate senescence, exemplified by mitochondrial dysfunction-associated senescence (MiDAS). MiDAS is characterized by impaired oxidative phosphorylation, reduced membrane potential, and altered NAD^+^ metabolism, and exhibits a distinct SASP profile lacking IL-1–dependent inflammatory components while retaining alternative cytokines (Wiley et al., 2016).

Environmental exposures further reinforce mitochondria-driven SIPS. Alcohol and cigarette smoke extract increase mitochondrial fission and mtROS, while impaired mitophagy promotes perinuclear accumulation of damaged mitochondria (Chen et al., 2017; Bonet-Ponce et al., 2015; Hara et al., 2013; Ahmad et al., 2015). Heavy metals such as hexavalent chromium induce mitochondrial respiratory chain dysfunction, elevate ROS, and activate p53-dependent senescence programs (Zhang Y. et al., 2016).

Collectively, SIPS highlights a mitochondria-centered model of senescence in which diverse stressors converge on mitochondrial dysfunction. Unlike replicative senescence, where telomere attrition is the primary trigger, SIPS is driven by acute mitochondrial perturbations that initiate and amplify senescence signaling through ROS production, metabolic disruption, and inflammatory reprogramming.

## 5 Mitochondria in oncogene-induced senescence

Oncogene-induced senescence (OIS) is a durable growth-arrest program triggered by aberrant oncogenic signaling, functioning as an intrinsic tumor-suppressive mechanism. A defining feature of OIS is early mitochondrial activation, characterized by increased mitochondrial mass, elevated mtDNA content, and enhanced ROS production (Mo et al., 2009). In oncogenic Ras-driven models, ROS generation is tightly regulated by NOX4, a constitutively active NADPH oxidase. NOX4-derived ROS initially support hyperproliferative signaling but subsequently trigger DNA damage responses (DDR) required for senescence establishment (Weyemi and Dupuy, 2012; Ogrunc et al., 2014). The NOX4–p22 complex is essential for this transition, as its disruption impairs DNA damage foci formation, p21 induction, senescence-associated heterochromatin foci (SAHF), and PML body assembly, positioning NOX4-dependent redox signaling as a key upstream driver of OIS (Weyemi et al., 2012).

As OIS progresses, mitochondrial dysfunction becomes more pronounced. Increased mitochondrial mass is coupled with impaired mitophagy, leading to accumulation of dysfunctional organelles (Korolchuk et al., 2017). These mitochondria exhibit reduced membrane potential, decreased ATP production, and activation of AMPK through p53/Rb-dependent pathways, collectively reinforcing stable growth arrest (Mo et al., 2009). Morphologically, mitochondria shift toward a hyperfused and elongated network, reflecting suppressed fission and enhanced fusion dynamics (Martini and Passos, 2023). In parallel, mitochondrial stress promotes cytosolic release of mtDNA, which activates the cGAS–STING pathway to drive SASP gene expression (Victorelli et al., 2023).

Calcium signaling represents another critical layer of mitochondrial regulation in OIS. Downregulation of TRPC3 promotes mitochondrial Ca^2+^ overload, enhancing inflammatory senescence phenotypes (Farfariello et al., 2022). ER-derived Ca^2+^ release via ITPR2 is transferred to mitochondria through the mitochondrial calcium uniporter (MCU), leading to membrane depolarization, increased ROS production, and reinforcement of senescence (Wiel et al., 2014). Additionally, upregulation of the sodium channel SCN9A elevates cytosolic Ca^2+^ levels, triggering Rb/E2F-dependent mitotic arrest and stabilizing the senescent state (Warnier et al., 2018).

Metabolic rewiring further supports mitochondrial dysfunction and sustains the senescence-associated secretory phenotype (SASP). Many OIS models enhance fatty acid oxidation and maintain high oxidative phosphorylation (OXPHOS) activity to meet the energetic demands of SASP production (Quijano et al., 2012). These seemingly contrasting observations likely reflect temporal and context-dependent metabolic adaptations during OIS. Early stages of OIS are frequently characterized by enhanced mitochondrial activity and elevated OXPHOS to support biosynthetic demands and SASP production. However, persistent oncogenic stress, impaired mitophagy, and progressive mitochondrial damage can reduce ATP-generating efficiency despite sustained or compensatory respiratory activity, resulting in decreased ATP production in established senescent cells. However, metabolic adaptations are highly context-dependent. RB-driven senescence promotes glycolytic gene expression, whereas Ras-driven OIS elevates NAMPT-dependent NAD^+^/NADH ratios to simultaneously support glycolysis and mitochondrial respiration (Takebayashi et al., 2015; Nacarelli et al., 2019). In contrast, certain epithelial models reduce glycolytic flux, with forced glycolysis enabling escape from senescence, while some fibroblast systems exhibit enhanced OXPHOS alongside suppressed glycolysis, effectively reversing the Warburg-like phenotype (Gitenay et al., 2014). BRAFV600E-induced senescence further exemplifies mitochondrial activation by stimulating pyruvate dehydrogenase (PDH), thereby increasing TCA cycle flux, mitochondrial respiration, and redox stress (Kaplon et al., 2013). Oncogenic stress also disrupts nucleotide metabolism, linking metabolic imbalance to mitochondrial dysfunction and cell cycle arrest. Ras activation alters metabolic flux by increasing fructose-1,6-bisphosphate and M2-PK activity while depleting nucleotide pools such as UTP and CTP, thereby impairing DNA replication and reinforcing senescence (Mazurek et al., 2001). Recent work by Marmisolle et al. (2025) further emphasizes the heterogeneity of senescence-associated metabolic remodeling. In contrast to metabolic adaptations observed in replicative senescence, oncogene-induced senescence exhibits distinct alterations in citrate metabolism and epigenetic regulation, suggesting that mitochondrial rewiring is highly dependent on the initiating senescence stimulus.

Collectively, OIS is defined by an early surge in mitochondrial activity followed by progressive mitochondrial dysfunction, with redox signaling, Ca^2+^ dynamics, and metabolic rewiring acting in concert to establish and stabilize the senescent state.

## 6 Mitochondria in therapy-induced senescence

Therapy-induced senescence (TIS) is a senescence-like growth arrest triggered by anticancer treatments, including chemotherapy, radiotherapy, and targeted therapies. TIS exhibits substantial phenotypic heterogeneity depending on the inducing agent, with diverse marker profiles across cell types, underscoring the need for multi-parameter identification beyond single markers such as SA-β-gal or p21 (Bojko et al., 2019). At the organelle level, TIS is characterized by early mitochondrial remodeling, including increased mitochondrial volume followed by lipid vesicle accumulation (Yamauchi et al., 2024; Bresci et al., 2023).

Mitochondrial dynamics in TIS commonly shift toward a hyperfused and elongated network, driven by dysregulated fission–fusion balance and impaired mitophagy (Martini and Passos, 2023). For example, temozolomide-induced senescence increases MFN1/2 expression, promoting mitochondrial fusion and reshaping ROS signaling, bioenergetics, and mitochondria–cytosol communication, which collectively regulate SASP production (Tarallo et al., 2024). Functionally, mitochondrial bioenergetics in TIS are highly context-dependent. Many treatments, including doxorubicin and oxidative stress, reduce oxygen consumption rate (OCR) and impair mitochondrial function, whereas direct mitochondrial damage can induce mitochondrial dysfunction-associated senescence (MiDAS) (Wiley et al., 2016). Cisplatin, for instance, targets mtDNA, disrupts mitochondrial homeostasis, and promotes ROS accumulation, driving senescence-like states (Kleih et al., 2019). Similarly, doxorubicin treatment elevates ROS and reduces mitochondrial membrane potential in both cancer and normal cells (Kleih et al., 2019; Song et al., 2012). In contrast, certain models such as melanoma display increased mitochondrial mass and enhanced oxidative phosphorylation (OXPHOS), highlighting metabolic heterogeneity in TIS (Martínez et al., 2019).

These mitochondrial alterations drive pronounced metabolic reprogramming. In breast cancer models, doxorubicin and ionizing radiation induce senescence accompanied by ROS-associated mitochondrial dysfunction and altered extracellular metabolite profiles (George et al., 2024). Radiation-induced senescence frequently promotes a shift toward glycolysis with AMPK activation, reflecting mitochondrial energy stress and leading to lactate secretion, extracellular acidification, and paracrine signaling that can influence neighboring cells (Liao et al., 2014). Similar metabolic rewiring is observed in irradiated colon cancer cells, where suppressed TCA cycle activity forces reliance on aerobic glycolysis with increased pyruvate and lactate accumulation (Nagineni et al., 2021).

Beyond metabolism, TIS reshapes mitochondrial control of apoptosis. Senescent cancer cells exhibit altered mitochondrial apoptotic priming, determined by the balance of BCL-2 family proteins (King et al., 2022; Chonghaile et al., 2011). Although overall apoptotic priming is reduced, TIS cells retain a form of “mitochondrial memory,” maintaining selective dependence on specific anti-apoptotic proteins (Menendez et al., 2025). BH3 profiling studies further demonstrate that these dependencies remain targetable, revealing mitochondria as a critical vulnerability that can be exploited using BH3 mimetics or immunosenolytic strategies (Menendez et al., 2025).

Collectively, TIS represents a highly heterogeneous senescence program in which therapeutic stress converges on mitochondrial remodeling. Changes in mitochondrial dynamics, bioenergetics, metabolism, and apoptotic priming integrate to determine both the stability of senescence and the therapeutic vulnerabilities of TIS cells.

## 7 Targeting mitochondria in senescence

Given that mitochondria lie at the core of multiple senescence programs, therapeutic strategies increasingly aim to modulate mitochondrial function to either restore cellular homeostasis or selectively eliminate harmful senescent cells. In the broader senescence field, anti-senescence interventions are commonly classified as senolytics and senomorphics. Within the context of mitochondrial-targeted interventions, the approaches discussed here broadly fall into two categories: (i) rejuvenation through restoration of mitochondrial function and (ii) senolytic elimination by exploiting mitochondrial vulnerabilities. Notably, rejuvenation-oriented strategies largely overlap conceptually with senomorphic interventions because they attenuate senescence-associated phenotypes without directly eliminating senescent cells.

Restoration of mitochondrial function has shown promise in reversing senescence-associated phenotypes. Mitochondrial transplantation into replicative or oxidative stress–induced senescent cells improves bioenergetics, reduces oxidative stress, and suppresses senescence markers such as SA-β-gal activity, NF-κB signaling, inflammatory cytokines, and p21/p16 expression (Noh et al., 2023). Similarly, small extracellular vesicles derived from young stem cells rejuvenate senescent mesenchymal stem cells by restoring mitochondrial dynamics via Drp1 activation and recovering stemness and immunomodulatory capacity (Peng et al., 2024).

Metabolic and pharmacological interventions further highlight the plasticity of mitochondrial regulation in senescence. Low-dose resveratrol delays oxidative stress–induced senescence by inducing adaptive ROS signaling, enhancing mitochondrial biogenesis, and reducing DNA damage (Mikuła-Pietrasik et al., 2012). Systemic interventions such as calorie restriction attenuate senescence-associated transcriptional programs and improve metabolic health, indicating that organismal metabolic rewiring can modulate senescence (Aversa et al., 2024). Additional strategies include NAD^+^ repletion (e.g., nicotinamide riboside), which activates mitochondrial stress responses and mitophagy to rejuvenate stem cells, CoQ_10_-mediated suppression of ROS–Akt/mTOR signaling, and NANOG-driven metabolic reprogramming through the proline–PYCR1/2–AMPK–Parkin axis (Zhang H. et al., 2016; Yang et al., 2021; Zhang et al., 2015; Choudhury et al., 2024).

Several additional interventions have emerged as promising approaches for restoring mitochondrial quality control through activation of autophagy and mitophagy pathways. Spermidine stimulates autophagy and improves mitochondrial homeostasis, whereas urolithin A enhances mitophagy and has demonstrated beneficial effects on muscle function and aging-associated phenotypes. Berberine has also been reported to improve mitochondrial quality control through AMPK-dependent signaling and mitophagy activation. Together, these findings further support the concept that restoration of mitochondrial turnover represents a promising strategy for mitigating senescence-associated dysfunction and promoting healthy aging (Ryu et al., 2016; Eisenberg et al., 2009).

Mitochondria-derived peptides (MDPs), including humanin and MOTS-c, add an additional regulatory layer by functioning as systemic signaling molecules. These peptides are upregulated in senescent cells, enhance mitochondrial respiration, and modulate SASP output via pathways such as JAK signaling, while also protecting certain cell types from oxidative stress and mitochondrial dysfunction (Kim et al., 2018; Sreekumar et al., 2016; Lee et al., 2015). This dual role positions MDPs as both biomarkers and therapeutic modulators of mitochondrial senescence states. Importantly, mitochondrial adaptations may also influence senolytic susceptibility in a stimulus-dependent manner. Replicative, stress-induced, oncogene-induced, and therapy-induced senescence exhibit distinct patterns of mitochondrial remodeling, bioenergetic dependency, and anti-apoptotic signaling. These differences may contribute to variability in sensitivity to BH3 mimetics and other mitochondria-targeted senolytic strategies, highlighting the need for senescence subtype-specific therapeutic approaches.

In contrast, senolytic strategies exploit mitochondrial dependencies that arise during senescence. Senescent cells frequently rely on specific anti-apoptotic BCL-2 family proteins, and this dependency—rather than expression level alone—predicts sensitivity to senolytics such as ABT-263 or dasatinib plus quercetin (MacDonald et al., 2025). For example, therapy-induced senescent melanoma cells exhibit BCL-xL addiction driven by reduced HRK and increased free BCL-xL, rendering them selectively vulnerable to BCL-xL inhibitors such as A-1331852, navitoclax, and DT2216 (Alcon et al., 2024). BH3 profiling further demonstrates that mitochondrial apoptotic priming—often established prior to full senescence—serves as a predictive biomarker for senolytic responsiveness, with high priming and low MCL-1 correlating with sensitivity (Jochems et al., 2025).

Beyond apoptotic signaling, structural and metabolic mitochondrial vulnerabilities can also be targeted. The mitochondria-directed agent MitoTam selectively eliminates senescent cells by exploiting their intrinsically low levels of adenine nucleotide translocase 2 (ANT2), which renders them hypersensitive to mitochondrial stress (Eliska Vacurova et al., 2024; Hubackova et al., 2019). Restoration of ANT2 rescues this sensitivity, identifying ANT2-dependent mitochondrial dysfunction as a key determinant of senolytic susceptibility. Collectively, these findings position mitochondria as a central therapeutic hub in senescence biology. Effective interventions will likely require integrated strategies that combine mitochondrial restoration with selective elimination, supported by functional biomarkers such as mitochondrial priming. Such approaches may enable precise, context-dependent modulation of senescence, minimizing pathological effects while preserving tissue homeostasis and regenerative capacity.

## 8 Discussion

Accumulating evidence indicates that mitochondria function as active orchestrators—rather than passive bystanders—of the senescence program. Mitochondrial alterations shape multiple layers of senescence biology, including redox signaling, metabolic rewiring, epigenetic regulation, and the inflammatory senescence-associated secretory phenotype (SASP).

A central emerging principle is the context dependency of mitochondrial regulation. Distinct senescence-inducing stimuli generate qualitatively different mitochondrial states that ultimately define senescent cell phenotypes. Replicative senescence is governed by a telomere–mitochondria feedback loop involving the p53–PGC-1 axis, whereas stress-induced and oncogene-induced senescence are characterized by rapid mitochondrial stress responses and stimulus-specific metabolic rewiring. Therapy-induced senescence introduces an additional layer of complexity, as senescent cancer cells often acquire unique mitochondrial dependencies that influence both treatment resistance and susceptibility to senolytic interventions.

This mitochondrial heterogeneity has important therapeutic implications. Approaches aimed at restoring mitochondrial quality control—such as enhancing mitophagy, NAD^+^ metabolism, or mitochondrial biogenesis—may rejuvenate aged tissues and stem cell function (Zhang H. et al., 2016; Yang et al., 2021). In contrast, senolytic strategies can selectively eliminate senescent cells by exploiting mitochondrial apoptotic priming and metabolic vulnerabilities (Yang et al., 2021).

Looking forward, a major priority will be to define mitochondrial signatures that distinguish functionally distinct senescent cell populations *in vivo*. The development of predictive tools—such as BH3 profiling to assess apoptotic priming—will be critical for stratifying senescence states and guiding targeted interventions (Jochems et al., 2025). Ultimately, integrating mitochondrial biology with senescence heterogeneity will enable the design of precision therapeutics that either modulate or selectively eliminate senescent cells, while preserving tissue homeostasis in aging, cancer, and degenerative diseases.
